# Performance and stability gain in zero-dimensional perovskite solar cells after >2 years when hybridized with silicon nanocrystals

**DOI:** 10.1039/c9na00516a

**Published:** 2019-11-08

**Authors:** Calum McDonald, Chengsheng Ni, Paul Maguire, Davide Mariotti, Vladimir Svrcek

**Affiliations:** Research Center for Photovoltaics, National Institute of Advanced Industrial Science and Technology (AIST) Central 2, Umezono 1-1-1 Tsukuba 305-8568 Japan calum.mcdonald@aist.go.jp; College of Resources and Environment, Southwest University Beibei Chongqing China; Nanotechnology & Integrated Bio-Engineering Centre (NIBEC), Ulster University Shore Road Newtownabbey BT37 0QB UK

## Abstract

We report highly stable zero-dimensional (CH_3_NH_3_)_3_Bi_2_I_9_ photovoltaic cells which demonstrate a 33% increase in performance after 2 years when hybridized with silicon nanocrystals (SiNCs). The natural oxidation of SiNCs is expected to consume radical species and improve the SiNC/(CH_3_NH_3_)_3_Bi_2_I_9_ interface and electronic coupling whilst also inhibiting defect-induced degradation.

## Abstract 2

We report highly stable zero-dimensional (CH_3_NH_3_)_3_Bi_2_I_9_ photovoltaic cells which demonstrate a 33% increase in performance after 2 years when hybridized with silicon nanocrystals (SiNCs).

## Graphical abstract

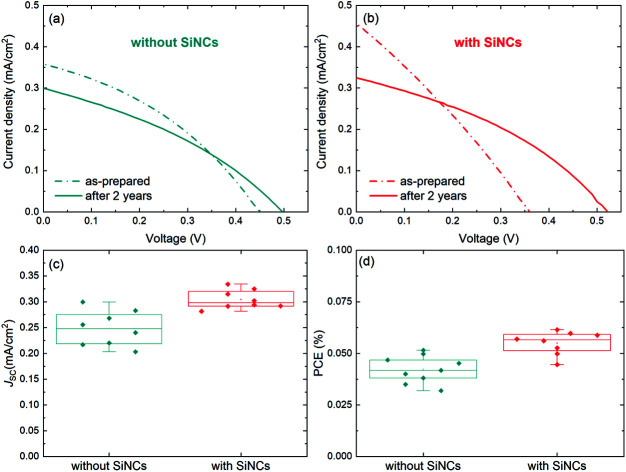

## Introduction

Organometal halide perovskites (OHPs) have demonstrated outstanding photovoltaic performance and have become a leading photovoltaic technology in less than a decade with power conversion efficiencies (PCEs) that now exceed 24%.^1^ Despite the remarkable PCEs demonstrated by OHP devices, there remain major concerns regarding their suitability for commercialisation,^2^ largely based on the poor stability of the OHP absorber, whether such devices can perform consistently over a period of decades and in harsh weather environments, and the ecological impact of Pb contamination. Although mixing different cations has led to improvements in the stability in recent years,^3^ degradation is still observed even when devices are encapsulated.^4^ Encapsulation is seemingly essential for OHPs and would significantly increase manufacturing costs. However, encapsulation is problematic since the low fracture energies of OHPs and thermal expansion mismatch between the perovskite and encapsulant cause delamination, particularly under thermal cycling.^4^ For several years there has been strong interest from the research community to solve the stability issue of OHPs. Notable approaches to improve the stability include reducing the dimensionality,^5^ forming double perovskites,^6^ forming all-inorganic perovskites^7^ and introducing nanocrystals into the perovskite structure.^8^ So far, the most impressive efficiencies and stabilities have been achieved by forming 2D sheets or 1D chains of OHPs where the OHP can be encapsulated by a long chain polymer.^9^

While attempts to improve the stability of lead-based OHPs have seen some success, the long-term outlook still remains unclear. On the other hand, starting with a perovskite material which shows intrinsic high stability, yet requires dedicated work towards improving its efficiency, is an alternative route towards low-cost, high efficiency photovoltaics with commercial appeal. Methylammonium bismuth iodide (MABI), with the chemical formula (CH_3_NH_3_)_3_Bi_2_I_9_, is an air-stable organic–inorganic hybrid material which can be considered a metal-deficient perovskite, *i.e.* AB_2/3_X_3_.^10^ In MABI, the 3D perovskite structure has been disconnected in all three dimensions resulting in a zero-dimensional network of face-sharing Bi_2_I_9_^3−^ bi-octahedra. These bi-octahedra are isolated and stabilised by MA^+^ cations (CH_3_NH_3_^+^). A 0D internal structure offers fascinating opportunities, such as the localisation of carriers on Bi_2_I_9_^3−^ clusters which has allowed the demonstration of carrier multiplication in MABI.^11^ Previous work has indicated that MABI is highly stable, attributed to the formation of a native oxide surface layer of BiOI or Bi_2_O_3_ which does not inhibit carrier extraction in solar cell devices.^10^ The high stability of MABI is further evidenced by the absence of hysteresis in MABI devices.^12^

MABI was initially prepared as a lead-free alternative to OHPs but has not replicated the monumental rise in efficiency of lead-based OHPs. To date, the best cells have efficiencies of approximately 1.64%.^13^ MABI must overcome several fundamental constraints in order to achieve higher stability, such as exciton dissociation, poor charge transport and carrier extraction issues.^14^ Whilst MABI has a large bandgap (∼2 eV), bandgap tuning *via* doping and ion substitution has been demonstrated to lower the bandgap to as low as 1.45 eV.^15–17^ Its high stability also makes it a promising top cell in a multi-junction device, whereby MABI can provide encapsulation for the less-stable bottom cell. The emerging field of indoor PVs also presents a development opportunity for MABI-based devices where the required ideal bandgap matches that of MABI.^18^ Furthermore, recently we showed the benefits of incorporation of femtosecond laser surface-engineered silicon nanocrystals (SiNCs) to form perovskites/SiNCS based hybrids that can increase the efficiency of low-dimensional perovskite solar cells.^12^

In this communication we report the results of the stability test of MABI solar cell devices with and without SiNCs after a period of more than 2 years. Our investigation demonstrates highly stable MABI solar cells which show enhanced stability when incorporated with SiNCs, resulting in an increase in the performance after >2 years. We discuss the mechanism in detail based on these results and demonstrate that the low-dimensional perovskite MABI is a highly stable photovoltaic material and should be further studied for photovoltaics, particularly through forming hybrids and doping.

## Results and discussion

**Fig. 1:**
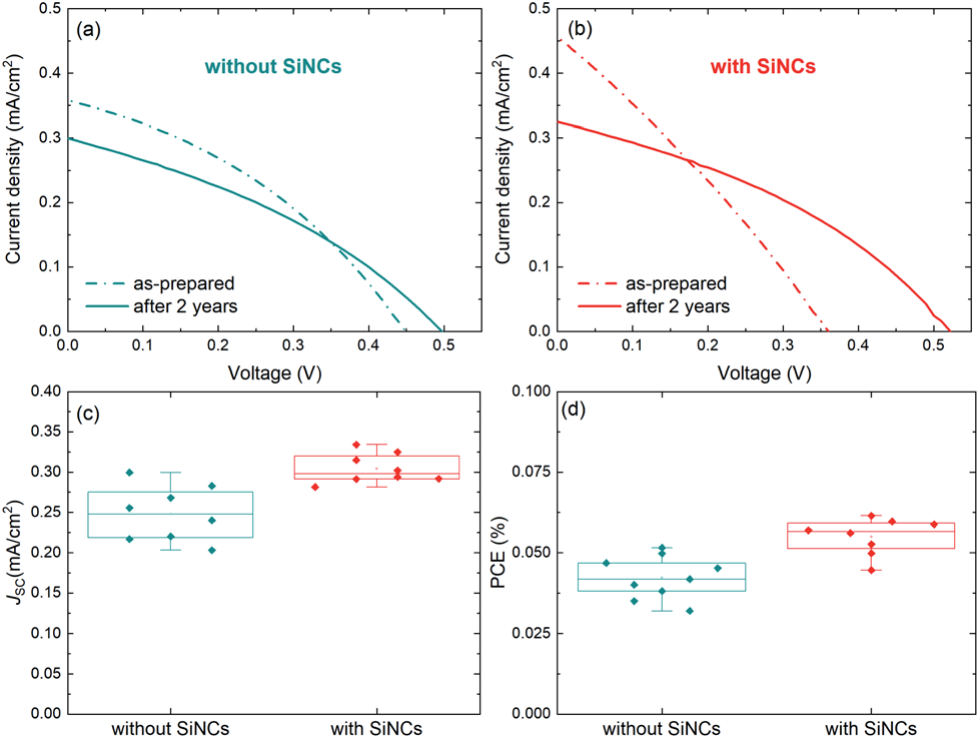
Comparison of the champion solar cell current density–voltage characteristics after fabrication and after 2 years for MABI solar cells (a) without SiNCs and (b) with SiNCs. Statistical analysis of the device performance after two years for (c) the short-circuit current density (*J*_SC_) and (d) the power conversion efficiency (PCE). The data in (c and d) are plotted as box and whisker plots as per the standard Tukey boxplot, where the “box” represents the respective quartiles below and above the mean, and the “whiskers” represent the lowest (highest) datum within a range defined by the interquartile range multiplied by a factor of 1.5, relative to the lower (upper) quartile.

The device structure was formed by indium-doped tin oxide (ITO)/compact TiO_2_/mesoporous TiO_2_/MABI/spiro-MeOTAD/Au (the fabrication details are reported in the Experimental details section). 8 MABI devices were fabricated without SiNCs and 8 MABI devices were fabricated with SiNCs. Here we use surface-engineered SiNCs with a mean diameter of 3.2 nm with a narrow size distribution.^19^ The SiNC surface is formed by a degree of oxidation along with Si–OH terminations (the full details are in the Experimental details section). Fig. 1 shows the performance of devices prepared and stored under open-air conditions recorded shortly after fabrication and after >2 years (738 days). The current density–voltage (*J*–*V*) characteristics of the champion MABI device without SiNCs (Fig. 1a) and with SiNCs (Fig. 1b) are shown. In both cases, there is a clear decrease in the short-circuit current density (*J*_SC_) after 2 years, which is far more pronounced for the hybrid devices. Both types of devices indicate an increase in the fill factor (FF) and in the open-circuit voltage (*V*_OC_), which is significantly noticeable for the hybrid devices. Fig. 1c and d show the statistical distributions and averages of the *J*_SC_ and PCE, respectively, for the devices after >2 years. Both the average *J*_SC_ and the PCE remained higher for the devices with SiNCs. SiNCs are expected to become partially oxidised within this time-frame as previously observed;^8^ however this does not seem to have had a dramatic impact on the device performance considering that the hybrid solar cells with SiNCs perform better overall even after >2 years.

**Fig. 2:**
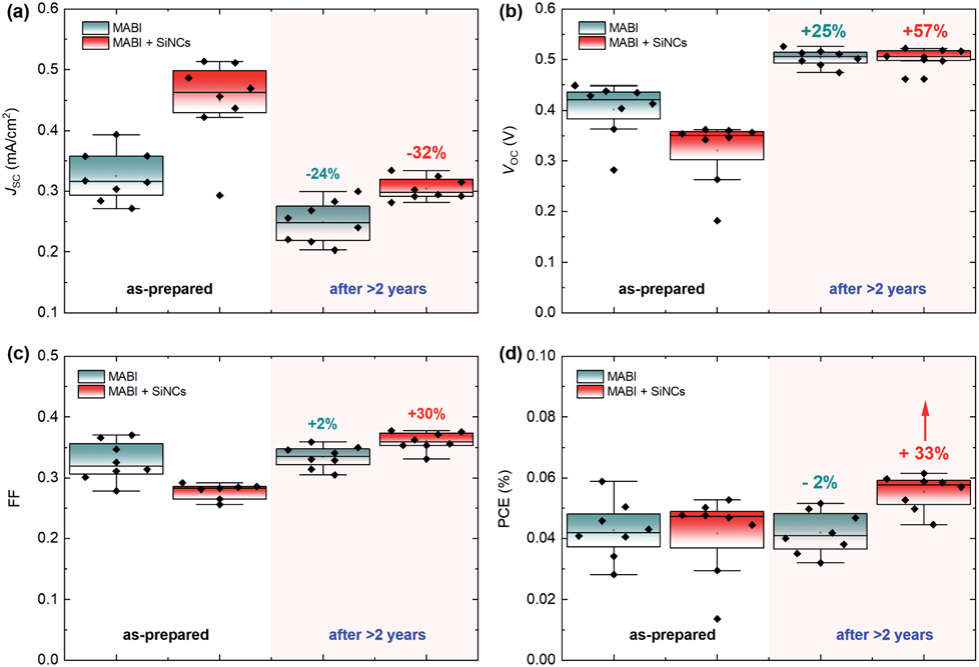
MABI device stability with and without SiNCs, as-prepared and after 2 years for (a) short-circuit current density (*J*_SC_), (b) open-circuit voltage (*V*_OC_), (c) fill-factor (FF) and (d) power conversion efficiency (PCE). The data are plotted as a box and whisker plot as per the standard Tukey boxplot, where the “box” represents the respective quartiles below and above the mean, and the “whiskers” represent the lowest (highest) datum within a range defined by the interquartile range multiplied by a factor of 1.5, relative to the lower (upper) quartile.

Fig. 2 shows the stability of MABI solar cells after a period of two years, both with and without SiNCs. The average values are plotted for each performance parameter with error bars representing standard deviation. The *J*_SC_ for devices both with and without SiNCs decreased considerably after 2 years, more so for the hybrid devices (Fig. 2a). Hybrid devices show a far higher initial *J*_SC_, which is however reduced after 2 years likely due to the oxidation of SiNCs. There was also a reduction in the *J*_SC_ for MABI-only devices, but less pronounced. The initial high *J*_SC_ for the hybrid devices was previously attributed to several possible factors introduced by the SiNCs: greater crystal quality of the MABI film, improved carrier transport, and/or a favourable exciton dissociation pathway *via* SiNCs.^12^ Following >2 years aging under ambient conditions, the *J*_SC_ for hybrid devices decreased to close to (but still higher than) the *J*_SC_ of the MABI-only devices. The corresponding standard deviation for the hybrid device was also reduced after 2 years. These results may suggest that the role played by the SiNCs in the photocurrent has been suppressed after oxidation; however, even after oxidation, hybrid devices still displayed higher *J*_SC_ values which we expect is largely due to the overall superior crystal quality of the hybrid layer.

Initially, MABI-only devices exhibit a mean (*x̄*) *V*_OC_ of 0.4 V, which is higher than that of the hybrid devices (*x̄* = 0.32 V). The generally low *V*_OC_ of both hybrid and intrinsic devices is likely due to high carrier recombination rates caused by surface traps at the interface between MABI and the transport layers. Recombination is further exacerbated in the hybrid devices due to a higher density of traps and defects at interfaces between MABI and the SiNCs resulting in the lower *V*_OC_. After some time (∼1 month), a BiO/Bi_2_O_3_ surface layer is expected to form^10^ and we assume the formation of the surface layer is completed in under 2 years for both types of devices as we observe the stabilisation of the *V*_OC_ at 0.5 V for both MABI and MABI/SiNC hybrid devices, with a very small standard deviation (Fig. 2b). This strongly suggests that a passivating surface layer has formed having the same properties and effects irrespective of the absence or presence of SiNCs. Previous work by Hoye *et al.* showed that a surface layer became noticeable only after ∼one month of exposure to air; this was identified by an increase in the O–Bi peak in the X-ray photoelectron spectra (XPS).^10^ XRD patterns revealed no changes after 13 days, with only slight changes to the patterns observed after 25 days suggesting the presence of a small amount of either Bi_2_O_3_ or BiOI, concurrent with the XPS results.^10^ The formation of the surface layer self-passivates the MABI layer and reduces the recombination rate at the trap states at the surface/grain boundaries. Since the final *V*_OC_ values for both MABI and MABI–SiNC hybrid devices are very close, we assume that SiNCs are either not present in the BiOI/Bi_2_O_3_ surface layer or that in any case do not play a role at the surface. These results, along with our observations, reveal that the surface layer can serve to improve the device *V*_OC_ likely *via* passivating surface defects and also providing protection against further degradation.

We also observed a large increase in the FF of hybrid devices which surpassed the FF of MABI-only devices after >2 years (Fig. 2c). The FF remained essentially unchanged for MABI-only devices with a small standard deviation. While we attribute the change in *V*_OC_ to the surface passivation of the MABI films, the large difference in the FF must be attributed to the presence of the SiNCs. When the device is initially fabricated, we expect the presence of SiNCs in the MABI layer to result in a higher density of defects at the interface between MABI and the SiNCs, and also somewhat at the grain boundaries caused by strain and incomplete crystallisation. While we previously observed an overall higher crystal quality in MABI/SiNC devices,^12^ this may not necessarily be true at the surface of the MABI film or at the grain boundaries. These defects result in the low initial FF of the hybrid devices due to higher recombination rates at the interface between MABI and the hole/electron transport layers, yet are passivated over time as the SiNCs are oxidised and chemically bond with the MABI structure.^8^

Considering the PCE, we observed an overall increase in the PCE of hybrid devices by 33%, demonstrating a significant increase in performance after >2 years. In the same time the PCE for MABI devices without SiNCs decreased slightly by 2%. The small decrease in the PCE of MABI-only devices by 2% demonstrates that MABI is intrinsically highly stable. Furthermore, the positive effect on the device performance is clearly attributed to the inclusion of SiNCs, where a higher *J*_SC_ than that of intrinsic MABI devices is retained after >2 years, along with significant gains in the *V*_OC_ and FF.

**Fig. 3:**
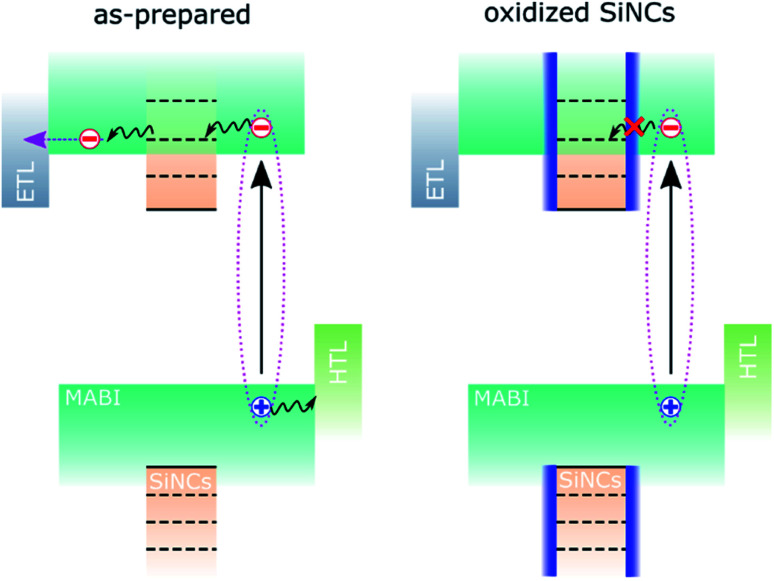
Schematic diagram of the band structure of MABI/SiNC hybrid devices, before and after oxidation of the SiNCs. ETL and HTL stand for the electron transport layer and hole transport layer, respectively.

We assume that the initial contribution of SiNCs is two-fold: improved MABI crystal quality and favourable exciton dissociation/extraction, as previously reported.^12^ Over time, the SiNCs become partially oxidised and an oxide shell is formed.^8^ The oxide shell likely inhibits the electronic interaction responsible for exciton dissociation and results in the reduction of the *J*_SC_. This is shown schematically in Fig. 3. We expect that defects at the interface between the MABI structure and the SiNCs may be passivated after oxidation, reducing trap-related recombination and leading to an increase in the FF and *V*_OC_.

## Conclusions

We have reported the long-term stability of MABI devices fabricated and stored under open-air conditions and observed stable performance for more than 2 years. While the MABI-only devices could retain the same PCE for 2 years, hybrid devices formed with MABI and SiNCs demonstrated an enhancement in the PCE with a 33% improvement. We propose the mechanisms responsible for the changes in the device performance parameters and attribute the increased performance and stability to a self-passivation of surface defects which is independent of the content of SiNCs. This passivating layer composed of either BiOI or Bi_2_O_3_ is likely responsible for the lower recombination rates at the surface *via* the reduction in surface trap density. Whilst the *J*_SC_ decreases in all cases, the final *J*_SC_ of hybrid devices remained higher than that of MABI-only devices, which we attribute to the superior initial crystal quality achieved *via* adding SiNCs. We also confirm that partial oxidation of the SiNCs does not have a negative impact on the overall device performance. This work demonstrates the potential of MABI to form highly stable solar cell devices. Moreover, we show that the incorporation of nanocrystals in hybrid devices can provide the opportunity to resolve stability issues of devices under ambient conditions.

## Experimental details

### SiNC synthesis and surface engineering

SiNCs were produced by electrochemical etching of p-type Si wafers in hydrochloric acid followed by mechanical removal of the resulting SiNC powder. 5 mg of SiNCs were dispersed in 3 mL of dimethylformamide and treated with a femtosecond laser to perform fragmentation and surface passivation following a similar method reported previously in detail.^19,20^ The SiNCs produced by electrochemical etching were characterized extensively in our previous work^8,21,22^ and following fs-laser processing the SiNCs have a mean diameter of 3.2 nm with a relatively narrow size distribution from 1 nm to 8 nm as confirmed by both transmission electron microscopy and Raman spectroscopy.^19^ The surface of the SiNCs after fs-laser processing is characterized by partial surface oxidation and OH-terminations.^19^ We should note that fs-laser surface engineering was previously carried out in water and ethanol;^19,20^ based on the resulting optical properties, we believe that fs-laser processing in DMF results in very similar SiNC characteristics.

### Solar cell device fabrication

16 solar cell devices in total, 8 devices with MABI alone and 8 hybrid devices composed of a MABI and SiNC composite layer, were produced. ITO glass slides were first treated with O_2_ plasma for 30 min. A compact TiO_2_ blocking layer was formed by first preparing a solution of titanium(iv) isopropoxide in ethanol and triethanolamine, which was spin coated (RPM = 5000, time = 30 s) and annealed at 400 °C for 2 hours. A mesoporous TiO_2_ layer was formed by diluting TiO_2_ nanoparticle paste (Dyesol 18-NRT) in ethanol in a 1 : 2 ratio of paste : ethanol and spin coating (RPM = 2,000, time = 30 s) and annealing at 400 °C for 2 hours. The MABI precursor solution was prepared by dissolving BiI_3_ (1.65 M) and CH_3_NH_3_I (2.475 M) in dimethylformamide. Hybrid devices were prepared by dissolving BiI_3_ (1.65 M) and CH_3_NH_3_I (2.475 M) in the SiNC-dimethylformamide solution; the SiNC-DMF solution was prepared as previously described. The MABI/MABI + SiNC precursor solution was stirred at 80 °C for 10 min and spin coated (RPM = 1500, time = 30 s). The film was then annealed at 100 °C for 30 min. The hole transport layer was prepared by dissolving 0.207 g 2,2′,7,7′-tetrakis[*N*,*N*-di(4-methoxyphenyl)amino]-9,9′-spirobifluorene (Spiro-MeOTAD) in 1 mL chlorobenzene and deposited by spin-coating (1500 RPM for 20 s). Gold metal contacts were deposited by thermal evaporation using a shadow mask. The resulting active area of the device was 0.04 cm^2^.

### Device storage conditions

Between measurements the devices were stored in air in the dark at room temperature and 50% relative humidity.

### Power conversion efficiency

The normalized solar spectrum AM1.5G was generated using a Wacom Electric Co. solar simulator (JIS, IEC standard conforming, CLASS AAA) calibrated to yield 100 mW cm^−2^ using an amorphous silicon (a-Si) reference cell. The electrical data were recorded using a Keithley 2400 source meter. The devices were tested in the voltage range of −0.1 V to 0.6 V without any pre-conditioning (*i.e.* without voltage soaking or light soaking).

## Conflicts of interest

There are no conflicts to declare.

## Supplementary Material
